# Telemedicine Use Among Adults With and Without Diagnosed Prediabetes or Diabetes, National Health Interview Survey, United States, 2021 and 2022

**DOI:** 10.5888/pcd21.240229

**Published:** 2024-11-14

**Authors:** Ibrahim Zaganjor, Ryan Saelee, Stephen Onufrak, Yoshihisa Miyamoto, Alain K. Koyama, Fang Xu, Kai McKeever Bullard, Meda E. Pavkov

**Affiliations:** 1 Division of Diabetes Translation, National Center for Chronic Disease Prevention and Health Promotion, Centers for Disease Control and Prevention, Atlanta, Georgia

## Abstract

We analyzed 2021 and 2022 National Health Interview Survey data to describe the prevalence of past 12-month telemedicine use among US adults with no prediabetes or diabetes diagnosis, diagnosed prediabetes, and diagnosed diabetes. In 2021 and 2022, telemedicine use prevalence was 34.1% and 28.2% among adults without diagnosed diabetes or prediabetes, 47.6% and 37.6% among adults with prediabetes, and 52.8% and 39.4% among adults with diabetes, respectively. Differences in telemedicine use were identified by region, urbanicity, insurance status, and education among adults with prediabetes or diabetes. Findings suggest that telemedicine use can be improved among select populations with prediabetes or diabetes.

**Summary**

**What is already known on this topic?**

In 2020, telemedicine use increased substantially due to the COVID-19 pandemic; however, nationally representative estimates of telemedicine use in recent years among US adults with prediabetes or diabetes are lacking.

**What is added by this report?**

This study’s results indicate that approximately one-third to one-half of adults diagnosed with prediabetes or diabetes used telemedicine in recent years. Results also demonstrate that among adults with these conditions, disparities in telemedicine use exist according to various sociodemographic characteristics.

**What are the implications for public health practice?**

This study’s findings suggest that disparities in telemedicine use can be reduced among select groups of adults living with prediabetes or diabetes.

## Objective

Telemedicine, the delivery of health care services at a distance, has a variety of potential benefits such as lower costs for patients, reduced strain on health care systems, and increased accessibility for select populations (eg, rural populations) (1). In particular, research suggests that telemedicine may improve diabetes-related clinical outcomes (2), enhancing the appeal for a wider application of telemedicine in the management and care of diabetes (3).

In 2021, an estimated 37.0% of US adults reported using telemedicine in the past 12 months, with use differing by several sociodemographic and geographic characteristics (4). However, nationally representative estimates of telemedicine use in recent years among US adults with prediabetes or diabetes are lacking. In this study, we aimed to describe the prevalence of past 12-month telemedicine use in 2021 and 2022 among US adults (aged 18 years or older) with no prediabetes or diabetes diagnosis, diagnosed prediabetes, and diagnosed diabetes. Additionally, since behavioral modifications related to the COVID-19 pandemic (eg, social distancing) likely influenced past 12-month telemedicine use in 2021 and 2022 differently, we also set out to identify characteristics associated with telemedicine use among each group in 2021 and 2022 separately to ascertain correlates persistently linked with use.

## Methods

We used 2021 and 2022 National Health Interview Survey (NHIS) data to conduct this analysis. The NHIS is a cross-sectional survey of the civilian, noninstitutionalized US population and has been described in detail previously (5,6). Self-reported history of diagnosed prediabetes or diabetes was used to identify 3 mutually exclusive populations: 1) no diabetes or prediabetes diagnosis; 2) diagnosed prediabetes; and 3) diagnosed diabetes. Adults were defined as having diagnosed prediabetes if they responded yes to the question, “Has a doctor or other health professional ever told you that you had prediabetes or borderline diabetes?” and no to the question, “Has a doctor or other health professional ever told you that you had diabetes?” Irrespective of a prediabetes diagnosis, adults who provided a positive response to the question specific to diabetes were categorized as having diabetes. Adults who responded no to both questions were considered to have no history of prediabetes or diabetes.

Past 12-month telemedicine use was defined by an affirmative response to the question, “In the past 12 months, have you had an appointment with a doctor, nurse, or other health professional by video or by phone?” For each year, we estimated crude prevalence and 95% CIs of past 12-month telemedicine use among all 3 populations and by select characteristics. We assessed differences in overall prevalence by year among each group using χ^2^ tests. We used logistic regression to calculate sex-, age-, and race and ethnicity–adjusted prevalence ratios (aPRs) to identify correlates of telemedicine use among each group. As a supplemental analysis, we repeated all analyses restricted to adults who saw a doctor or health professional within the past 12 months to describe telemedicine use patterns among adults with health care–seeking behaviors. We used SAS-callable SUDAAN (version 11.0.1, RTI International) to account for NHIS’s complex survey design.

## Results

In 2021 and 2022, the crude prevalence of telemedicine use in the past 12 months was, respectively, 34.1% and 28.2% among adults without diagnosed prediabetes or diabetes, 47.6% and 37.6% among adults with diagnosed prediabetes, and 52.8% and 39.4% among those with diagnosed diabetes (Figure). Across all 3 groups, telemedicine use prevalence decreased significantly between 2021 and 2022 (Figure). Among people diagnosed with diabetes, those with higher educational attainment were more likely to use telemedicine in both 2021 and 2022, whereas those who lacked insurance, lived in the Midwest or the South, or lived outside of large central or fringe metro areas were consistently less likely to use telemedicine (Table 1a and Table 1b). Among adults diagnosed with prediabetes, women and those with higher educational attainment were more likely to use telemedicine in the past 12 months, whereas adults without insurance and those living in nonmetropolitan areas, the Midwest, and the South were less likely to use telemedicine during both years. Consistent differences in telemedicine use were observed by sex, race and ethnicity, education, family income, insurance status, urbanicity, and region among adults with no prediabetes or diabetes diagnosis (Table 1a and Table 1b).

**Figure:**
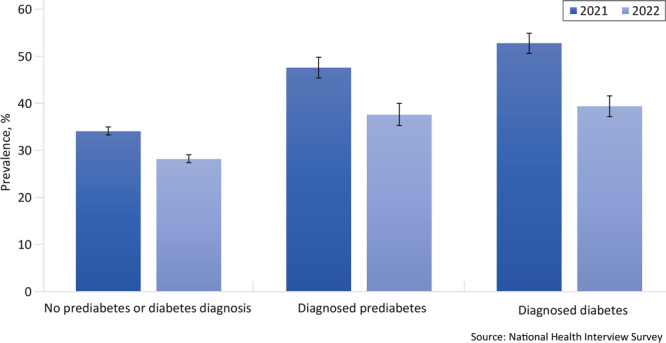
Unadjusted prevalence of telemedicine use in the past 12 months among adults with and without diagnosed prediabetes or diabetes. Prevalence (%) and associated 95% CIs are weighted; error bars indicate 95% CIs. For each population, differences between 2021 and 2022 were significant (all *P* < .05). Source: National Health Interview Survey, 2021 and 2022.

**Table:** 

Diagnosis	2021	2022
	% (95% CI)	
No diagnosed prediabetes or diabetes	34.1 (33.3–35.0)	28.2 (27.4–29.1)
Diagnosed prediabetes	47.6 (45.4–49.8)	37.6 (35.3–40.0)
Diagnosed diabetes	52.8 (50.6–54.9)	39.4 (37.2–41.6)

**Table 1a:** Prevalence of Telemedicine Use in the Past 12 Months Among Adults With and Without Diagnosed Prediabetes or Diabetes: National Health Interview Survey, United States, 2021^a^^,^ b

Characteristic	No prediabetes or diabetes diagnosis (n = 23,527)^c^		Diagnosed prediabetes (n = 2,542)		Diagnosed diabetes (n = 3,096)	
	Unadjusted % (95% CI)	aPR (95% CI)	Unadjusted % (95% CI)	aPR (95% CI)	Unadjusted % (95% CI)	aPR (95% CI)
**Overall**	34.1 (33.3–35.0)	NA	47.6 (45.4–49.8)	NA	52.8 (50.6–54.9)	NA
**Sex**						
Male	28.0 (26.9–29.0)	1 [Ref]	44.6 (41.0–48.2)	1 [Ref]	52.5 (49.6–55.5)	1 [Ref]
Female	39.9 (38.7–41.1)	1.4 (1.4–1.5)^d^	49.9 (46.9–52.9)	1.1 (1.0–1.3)^d^	53.0 (50.0–56.0)	1.0 (0.9–1.1)
**Age, y**						
18–44	31.5 (30.3–32.6)	1 [Ref]	46.3 (41.2–51.5)	1 [Ref]	58.3 (51.6–64.6)	1 [Ref]
45**–**64	35.0 (33.7–36.5)	1.1 (1.0–1.1)^d^	49.1 (45.7–52.6)	1.1 (0.9–1.2)	52.7 (49.3–56.0)	0.9 (0.8–1.0)
≥65	40.3 (38.7–41.9)	1.2 (1.1–1.3)^d^	46.3 (42.9–49.8)	1.0 (0.9–1.1)	51.8 (48.8–54.8)	0.9 (0.8–1.0)
**Race and ethnicity**						
White, non-Hispanic	36.9 (35.9–37.9)	1 [Ref]	48.7 (45.9–51.5)	1 [Ref]	52.5 (49.7–55.2)	1 [Ref]
Black, non-Hispanic	28.3 (26.0–30.8)	0.8 (0.7–0.8)^d^	43.5 (37.6–49.5)	0.9 (0.7–1.0)	52.3 (46.8–57.8)	1.0 (0.9–1.1)
Hispanic	28.7 (27.1–30.4)	0.8 (0.8–0.9)^d^	46.0 (40.2–51.9)	0.9 (0.8–1.1)	53.2 (47.4–58.9)	1.0 (0.9–1.1)
Other **e**	30.9 (28.5–33.5)	0.9 (0.8–0.9)^d^	49.6 (42.5–56.7)	1.0 (0.9–1.2)	54.5 (46.9–61.9)	1.1 (0.9–1.2)
**Education**						
No high school diploma or GED	23.5 (21.2–25.8)	1 [Ref]	40.4 (32.9–48.4)	1 [Ref]	44.7 (39.6–50.0)	1 [Ref]
High school diploma or GED	26.6 (25.3–28.0)	1.1 (1.0–1.2)^d^	40.1 (35.8–44.6)	1.0 (0.8–1.3)	49.7 (45.7–53.6)	1.1 (1.0–1.3)
Some college	35.9 (34.5–37.4)	1.5 (1.3–1.6)^d^	49.7 (45.6–53.8)	1.2 (1.0–1.5)^d^	55.0 (51.3–58.5)	1.3 (1.1–1.4)^d^
Bachelor's degree or higher	40.9 (39.7–42.2)	1.7 (1.5–1.9)^d^	54.5 (50.6–58.3)	1.4 (1.1–1.7)^d^	61.1 (56.7–65.3)	1.4 (1.2–1.6)^d^
**Family income, % FPL^f^**						
<100	29.6 (27.4–32.0)	1 [Ref]	45.4 (38.3–52.7)	1 [Ref]	46.7 (40.8–52.6)	1 [Ref]
100 to <200	28.7 (27.0–30.5)	1.0 (0.9–1.1)	41.5 (36.0–47.3)	0.9 (0.8–1.1)	48.2 (43.6–52.7)	1.0 (0.9–1.2)
200 to <400	32.3 (30.9–33.7)	1.1 (1.0–1.2)	46.9 (42.7–51.2)	1.0 (0.9–1.3)	54.0 (50.2–57.8)	1.2 (1.0–1.4)^d^
≥400	38.2 (37.1–39.4)	1.3 (1.2–1.4)^d^	51.3 (47.8–54.9)	1.1 (1.0–1.4)	57.6 (53.7–61.4)	1.3 (1.1–1.5)^d^
**Health insurance^g^**						
Private	36.1 (35.1–37.1)	1 [Ref]	48.6 (45.6–51.7)	1 [Ref]	52.9 (49.8–55.9)	1 [Ref]
Public only	39.2 (37.5–40.8)	1.1 (1.0–1.1)^d^	50.5 (46.5–54.5)	1.1 (1.0–1.2)	55.9 (52.7–59.0)	1.1 (1.0–1.2)
Uninsured	12.4 (10.8–14.1)	0.4 (0.3–0.4)^d^	22.7 (15.3–32.3)	0.5 (0.3–0.7)^d^	30.2 (22.1–39.8)	0.5 (0.4–0.7)^d^
**Urban–rural residence**						
Large central metro	37.2 (35.7–38.7)	1 [Ref]	51.3 (47.2–55.5)	1 [Ref]	59.2 (55.1–63.2)	1 [Ref]
Large fringe metro	37.6 (35.9–39.3)	0.9 (0.9–1.0)	51.3 (46.7–55.7)	1.0 (0.9–1.1)	54.9 (50.6–59.2)	0.9 (0.8–1.0)
Medium and small metro	31.6 (30.1–33.0)	0.8 (0.7–0.8)^d^	44.4 (40.6–48.3)	0.8 (0.8–1.0)^d^	49.3 (45.4–53.2)	0.8 (0.7–0.9)^d^
Nonmetropolitan	26.2 (24.2–28.3)	0.6 (0.6–0.7)^d^	39.8 (34.4–45.5)	0.7 (0.6–0.9)^d^	44.9 (39.6–50.3)	0.7 (0.6–0.8)^d^
**US Census region**						
West	38.3 (36.6–40.1)	1 [Ref]	55.4 (51.1–59.7)	1 [Ref]	67.1 (62.6–71.2)	1 [Ref]
Northeast	37.5 (35.6–39.5)	0.9 (0.9–1.0)	50.1 (44.6–55.7)	0.9 (0.8–1.0)	55.4 (49.7–61.0)	0.8 (0.7–0.9)^d^
Midwest	30.7 (28.8–32.6)	0.8 (0.7–0.8)^d^	46.8 (42.1–51.5)	0.8 (0.7–0.9)^d^	45.6 (41.4–49.9)	0.7 (0.6–0.7)^d^
South	31.7 (30.3–33.1)	0.8 (0.8–0.9)^d^	41.5 (38.1–44.9)	0.7 (0.7–0.8)^d^	48.3 (45.0–51.6)	0.7 (0.6–0.8)^d^

Abbreviations: aPR, adjusted prevalence ratio; FPL, federal poverty level; GED, general educational development certificate; NA, not available.

a Sample sizes (n) are unweighted. Prevalence (%) and associated 95% CIs are weighted and crude. aPRs were estimated using predictive marginal proportions from logistic regression models controlling for age, sex, and race and ethnicity.

b Telemedicine use in the past 12 months was based on a positive response to the survey question, “In the past 12 months, have you had an appointment with a doctor, nurse, or other health professional by video or by phone?”

c Diagnosed prediabetes was based on a positive response to the survey question, “Has a doctor or other health professional ever told you that you had prediabetes or borderline diabetes?” and a negative response to the survey question, “Has a doctor or other health professional ever told you that you had diabetes?” Diagnosed diabetes was based on a positive response to the survey question, “Has a doctor or other health professional ever told you that you had diabetes?” irrespective of a prediabetes diagnosis. Adults who responded no to both survey questions were considered to have no prediabetes or diabetes diagnosis. Adults missing complete prediabetes and diabetes diagnosis information were excluded.

d *P* < .05.

e “Other” category is composed of people who identified as non-Hispanic Asian, non-Hispanic American Indian or Alaska Native, other single race, or multiple races.

f Family income was imputed when missing. Family income was reported as a percentage of the FPL based on annual weighted average thresholds published by the US Census Bureau.

g “Private” is adults who reported having any private insurance plan. “Public only” is adults who did not have any private coverage but who reported being covered under Medicaid, Medicare, a state-sponsored health plan, other government program, or military coverage. “Uninsured” is adults who did not report being covered under private health insurance, Medicare, Medicaid, a state-sponsored health plan, other government program, or military coverage.

**Table 1b:** Prevalence of Telemedicine Use in the Past 12 Months Among Adults With and Without Diagnosed Prediabetes or Diabetes: National Health Interview Survey, United States, 2022^a^^,^ b

Characteristic	No prediabetes or diabetes diagnosis (n = 21,775)^c^		Diagnosed prediabetes (n = 2,659)		Diagnosed diabetes (n = 2,905)	
	Unadjusted % (95% CI)	aPR (95% CI)	Unadjusted % (95% CI)	aPR (95% CI)	Unadjusted % (95% CI)	aPR (95% CI)
**Overall**	28.2 (27.4–29.1)	NA	37.6 (35.3–40.0)	NA	39.4 (37.2–41.6)	NA
**Sex**						
Male	24.0 (23.0–25.1)	1 [Ref]	34.2 (31.0–37.5)	1 [Ref]	37.5 (34.5–40.6)	1 [Ref]
Female	32.2 (31.0–33.3)	1.3 (1.3–1.4)^d^	40.5 (37.4–43.7)	1.2 (1.0–1.3)^d^	41.4 (38.4–44.4)	1.1 (1.0–1.2)
**Age, y**						
18**–**44	27.9 (26.8–29.1)	1 [Ref]	40.0 (34.8–45.5)	1 [Ref]	45.8 (38.8–52.9)	1 [Ref]
45**–**64	28.8 (27.5–30.2)	1.0 (0.9–1.1)	37.7 (34.4–41.2)	0.9 (0.8–1.1)	42.3 (38.9–45.8)	0.9 (0.8–1.1)
≥65	28.1 (26.7–29.6)	0.9 (0.9–1.0)	35.7 (32.5–39.0)	0.9 (0.8–1.1)	35.1 (32.4–37.9)	0.8 (0.6–0.9)^d^
**Race and ethnicity**						
White, non-Hispanic	30.2 (29.2–31.2)	1 [Ref]	37.3 (34.4–40.3)	1 [Ref]	39.4 (36.8–42.0)	1 [Ref]
Black, non-Hispanic	24.2 (22.1–26.6)	0.8 (0.7–0.9)^d^	35.5 (30.2–41.1)	0.9 (0.8–1.1)	39.8 (34.6–45.2)	1.0 (0.8–1.1)
Hispanic	24.1 (22.4–25.9)	0.8 (0.7–0.9)^d^	37.8 (32.2–43.7)	1.0 (0.8–1.2)	36.7 (31.7–42.1)	0.9 (0.8–1.1)
Other **e**	26.9 (24.4–29.5)	0.9 (0.8–1.0)^d^	41.8 (35.1–48.9)	1.1 (0.9–1.3)	44.4 (36.7–52.3)	1.1 (0.9–1.3)
**Education**						
No high school diploma or GED	19.7 (17.5–22.3)	1 [Ref]	27.6 (21.6–34.7)	1 [Ref]	27.4 (22.7–32.7)	1 [Ref]
High school diploma or GED	21.6 (20.2–23.0)	1.1 (0.9–1.2)	30.7 (26.6–35.2)	1.2 (0.9–1.5)	35.7 (32.1–39.6)	1.3 (1.1–1.7)^d^
Some college	29.5 (28.1–31.0)	1.4 (1.3–1.6)^d^	39.8 (36.0–43.6)	1.5 (1.2–1.9)^d^	44.4 (40.4–48.5)	1.6 (1.3–2.0)^d^
Bachelor's degree or higher	34.6 (33.4–35.9)	1.7 (1.5–1.9)^d^	45.8 (41.9–49.7)	1.7 (1.3–2.2)^d^	49.0 (44.6–53.3)	1.8 (1.5–2.2)^d^
**Family income, % FPL^f^**						
<100	23.9 (21.7–26.2)	1 [Ref]	42.2 (35.5–49.1)	1 [Ref]	39.6 (33.9–45.6)	1 [Ref]
100 to <200	23.6 (22.1–25.3)	1.0 (0.9–1.1)	34.6 (29.6–40.0)	0.8 (0.7–1.0)	35.4 (31.0–40.0)	0.9 (0.7–1.1)
200 to <400	25.7 (24.4–27.1)	1.1 (1.0–1.2)	35.2 (31.4–39.2)	0.9 (0.7–1.1)	38.0 (34.3–41.9)	1.0 (0.8–1.2)
≥400	32.4 (31.2–33.5)	1.3 (1.2–1.5)^d^	39.8 (36.4–43.2)	1.0 (0.8–1.2)	43.6 (39.8–47.5)	1.1 (0.9–1.4)
**Health insurance^g^**						
Private	30.1 (29.1–31.2)	1 [Ref]	36.8 (34.0–39.7)	1 [Ref]	41.1 (38.2–44.1)	1 [Ref]
Public only	31.2 (29.7–32.7)	1.1 (1.0–1.1)^d^	42.2 (38.2–46.3)	1.2 (1.0–1.3)^d^	39.8 (36.6–43.1)	1.0 (0.9–1.1)
Uninsured	10.1 (8.6–11.8)	0.3 (0.3–0.4)^d^	20.7 (14.2–29.2)	0.5 (0.4–0.7)^d^	15.6 (9.2–25.2)	0.3 (0.2–0.6)^d^
**Urban–rural residence**						
Large central metro	32.2 (30.8–33.7)	1 [Ref]	41.7 (37.4–46.1)	1 [Ref]	43.9 (39.6–48.3)	1 [Ref]
Large fringe metro	31.1 (29.4–32.8)	0.9 (0.9–1.0)^d^	39.6 (35.3–44.0)	0.9 (0.8–1.1)	46.1 (41.5–50.8)	1.0 (0.9–1.2)
Medium and small metro	25.5 (24.1–27.0)	0.7 (0.7–0.8)^d^	37.8 (33.5–42.3)	0.9 (0.8–1.0)	36.5 (33.3–39.9)	0.8 (0.7–0.9)^d^
Nonmetropolitan	19.4 (17.3–21.7)	0.5 (0.5–0.6)^d^	24.5 (19.7–30.0)	0.6 (0.4–0.7)^d^	28.4 (23.7–33.6)	0.6 (0.5–0.7)^d^
**US Census region**						
West	34.0 (31.9–36.1)	1 [Ref]	46.9 (41.7–52.1)	1 [Ref]	45.3 (40.0–50.6)	1 [Ref]
Northeast	33.3 (31.5–35.2)	0.9 (0.9–1.0)	37.6 (32.6–42.9)	0.8 (0.7–0.9)^d^	44.0 (38.4–49.7)	1.0 (0.8–1.2)
Midwest	24.6 (22.8–26.4)	0.7 (0.6–0.7)^d^	33.4 (28.8–38.4)	0.7 (0.6–0.8)^d^	38.7 (34.9–42.6)	0.8 (0.7–1.0)^d^
South	24.2 (22.8–25.5)	0.7 (0.6–0.8)^d^	32.9 (29.4–36.6)	0.7 (0.6–0.8)^d^	35.2 (31.9–38.6)	0.8 (0.7–0.9)^d^

Abbreviations: aPR, adjusted prevalence ratio; FPL, federal poverty level; GED, general educational development certificate; NA, not available.

a Sample sizes (n) are unweighted. Prevalence (%) and associated 95% CIs are weighted and crude. Adjusted prevalence ratios (aPR) were estimated using predictive marginal proportions from logistic regression models controlling for age, sex, and race and ethnicity.

b Telemedicine use in the past 12 months is based on a positive response to the survey question, “In the past 12 months, have you had an appointment with a doctor, nurse, or other health professional by video or by phone?”

c Diagnosed prediabetes was based on a positive response to the survey question, “Has a doctor or other health professional ever told you that you had prediabetes or borderline diabetes?” and a negative response to the survey question, “Has a doctor or other health professional ever told you that you had diabetes?” Diagnosed diabetes was based on a positive response to the survey question, “Has a doctor or other health professional ever told you that you had diabetes?” irrespective of a prediabetes diagnosis. Adults who responded no to both survey questions were considered to have no prediabetes or diabetes diagnosis. Adults missing complete prediabetes and diabetes diagnosis information were excluded.

d *P* < .05.

e “Other” category is composed of people who identified as non-Hispanic Asian, non-Hispanic American Indian or Alaska Native, other single race, or multiple races.

f Family income was imputed when missing. Family income was reported as a percentage of the FPL based on annual weighted average thresholds published by the US Census Bureau.

g “Private” is adults who reported having any private insurance plan. “Public only” is adults who did not have any private coverage but who reported being covered under Medicaid, Medicare, a state-sponsored health plan, other government program, or military coverage. “Uninsured” is adults who did not report being covered under private health insurance, Medicare, Medicaid, a state-sponsored health plan, other government program, or military coverage.

In the supplemental analysis restricted to adults who saw a doctor or health professional within the past 12 months, the prevalence of telemedicine use in 2021 and 2022, respectively, was 39.9% and 32.4% among adults without diagnosed prediabetes or diabetes, 49.8% and 39.6% among adults diagnosed with prediabetes only, and 53.3% and 39.9% among adults diagnosed with diabetes (Table 2a and Table 2b). Correlates of telemedicine use remained generally similar among these 3 populations of interest.

**Table 2a:** Prevalence of Past 12-Month Telemedicine Use Among Adults With and Without Diagnosed Prediabetes or Diabetes Who Saw a Doctor or Health Professional Within the Past 12 Months: National Health Interview Survey, United States, 2021^a^^,^ b ^,^ c

Characteristic	No prediabetes or diabetes diagnosis (n = 19,106)^d^		Diagnosed prediabetes (n = 2,336)		Diagnosed diabetes (n = 2,989)	
	Unadjusted % (95% CI)	aPR (95% CI)	Unadjusted % (95% CI)	aPR (95% CI)	Unadjusted % (95% CI)	aPR (95% CI)
**Overall**	39.9 (38.9–40.8)	NA	49.8 (47.4–52.1)	NA	53.3 (51.1–55.5)	NA
**Sex**						
Male	34.5 (33.3–35.8)	1 [Ref]	47.4 (43.8–51.1)	1 [Ref]	53.3 (50.3–56.2)	1 [Ref]
Female	44.2 (43.0–45.5)	1.3 (1.2–1.3)^e^	51.5 (48.3–54.8)	1.1 (1.0–1.2)	53.4 (50.4–56.4)	1.0 (0.9–1.1)
**Age, y**						
18**–**44	38.4 (37.1–39.8)	1 [Ref]	51.6 (45.9–57.3)	1 [Ref]	59.9 (53.0–66.4)	1 [Ref]
45**–**64	40.5 (38.9–42.1)	1.0 (1.0–1.1)	51.6 (47.9–55.4)	1.0 (0.9–1.2)	53.5 (50.1–57.0)	0.9 (0.8–1.0)
≥65	42.4 (40.8–44.0)	1.1 (1.0–1.1)^e^	46.4 (42.8–50.0)	0.9 (0.8–1.0)	51.8 (48.8–54.8)	0.9 (0.8–1.0)^e^
**Race and ethnicity**						
White, non-Hispanic	42.2 (41.2–43.3)	1 [Ref]	49.7 (46.8–52.6)	1 [Ref]	52.9 (50.2–55.7)	1 [Ref]
Black, non-Hispanic	31.7 (29.1–34.4)	0.8 (0.7–0.8)^e^	46.6 (40.4–52.9)	0.9 (0.8–1.1)	53.9 (48.3–59.4)	1.0 (0.9–1.1)
Hispanic	36.2 (34.2–38.4)	0.9 (0.8–0.9)^e^	50.0 (43.6–56.4)	1.0 (0.8–1.1)	53.0 (47.0–59.0)	1.0 (0.9–1.1)
Other^f^	38.5 (35.4–41.7)	0.9 (0.8–1.0)^e^	54.7 (47.4–61.8)	1.1 (0.9–1.3)	55.4 (47.3–63.1)	1.1 (0.9–1.2)
**Education**						
No high school diploma or GED	29.9 (27.1–32.9)	1 [Ref]	41.5 (33.3–50.3)	1 [Ref]	45.1 (39.8–50.5)	1 [Ref]
High school diploma or GED	32.0 (30.4–33.7)	1.1 (1.0–1.2)	41.6 (37.1–46.4)	1.0 (0.8–1.3)	49.9 (45.8–53.9)	1.1 (1.0–1.3)
Some college	41.5 (39.9–43.2)	1.3 (1.2–1.5)^e^	52.9 (48.5–57.2)	1.3 (1.0–1.6)^e^	55.5 (51.8–59.2)	1.2 (1.1–1.4)^e^
Bachelor's degree or higher	46.3 (44.9–47.7)	1.5 (1.3–1.7)^e^	56.3 (52.5–60.1)	1.4 (1.1–1.7)^e^	61.9 (57.4–66.1)	1.4 (1.2–1.6)^e^
**Family income, % FPL^g^**						
<100	35.6 (33.0–38.4)	1 [Ref]	47.0 (39.5–54.7)	1 [Ref]	46.7 (40.7–52.8)	1 [Ref]
100 to <200	34.9 (32.9–37.0)	1.0 (0.9–1.1)	45.9 (39.9–52.0)	1.0 (0.8–1.2)	48.3 (43.7–52.9)	1.0 (0.9–1.2)
200 to <400	38.2 (36.6–39.8)	1.1 (1.0–1.1)	49.1 (44.6–53.7)	1.1 (0.9–1.3)	54.8 (50.9–58.7)	1.2 (1.0–1.4)^e^
≥400	43.4 (42.1–44.7)	1.2 (1.1–1.3)^e^	52.6 (49.0–56.1)	1.1 (1.0–1.4)	58.3 (54.3–62.1)	1.3 (1.1–1.5)^e^
**Health insurance^h^**						
Private	40.9 (39.8–42.1)	1 [Ref]	50.2 (47.1–53.3)	1 [Ref]	53.0 (50.0–56.0)	1 [Ref]
Public only	42.7 (40.9–44.5)	1.1 (1.0–1.1)^e^	51.9 (47.7–56.1)	1.1 (1.0–1.2)	56.4 (53.2–59.6)	1.1 (1.0–1.2)^e^
Uninsured	20.0 (17.4–22.9)	0.5 (0.5–0.6)^e^	29.6 (19.6–42.1)	0.6 (0.4–0.8)^e^	32.7 (23.7–43.1)	0.6 (0.4–0.8)^e^
**Urban–rural residence**						
Large central metro	44.4 (42.7–46.2)	1 [Ref]	54.9 (50.5–59.3)	1 [Ref]	59.9 (55.7–63.9)	1 [Ref]
Large fringe metro	42.7 (40.8–44.6)	0.9 (0.9–1.0)^e^	53.9 (49.3–58.5)	1.0 (0.9–1.1)	56.0 (51.5–60.4)	0.9 (0.8–1.0)
Medium and small metro	37.0 (35.4–38.6)	0.8 (0.7–0.8)^e^	45.9 (41.8–50.1)	0.8 (0.7–0.9)^e^	49.5 (45.5–53.4)	0.8 (0.7–0.9)^e^
Nonmetropolitan	30.6 (28.4–33.0)	0.6 (0.6–0.7)^e^	39.9 (34.7–45.3)	0.7 (0.6–0.8)^e^	45.3 (39.7–50.9)	0.7 (0.6–0.8)^e^
**US Census region**						
West	46.4 (44.4–48.4)	1 [Ref]	59.8 (55.1–64.4)	1 [Ref]	67.5 (62.7–71.9)	1 [Ref]
Northeast	42.7 (40.5–45.0)	0.9 (0.8–1.0)^e^	51.6 (45.6–57.6)	0.9 (0.7–1.0)^e^	56.5 (50.7–62.2)	0.8 (0.7–0.9)^e^
Midwest	35.7 (33.6–37.8)	0.7 (0.7–0.8)^e^	47.9 (43.0–52.8)	0.8 (0.7–0.9)^e^	46.1 (41.9–50.4)	0.7 (0.6–0.7)^e^
South	36.9 (35.3–38.4)	0.8 (0.7–0.8)^e^	43.3 (39.7–46.9)	0.7 (0.6–0.8)^e^	49.1 (45.8–52.4)	0.7 (0.6–0.8)^e^

Abbreviations: aPR, adjusted prevalence ratio; FPL, federal poverty level; GED, general educational development certificate; NA, not available.

a Sample sizes (n) are unweighted. Prevalence (%) and associated 95% CIs are weighted and crude. Adjusted prevalence ratios (aPR) were estimated using predictive marginal proportions from logistic regression models controlling for age, sex, and race and ethnicity.

b Telemedicine use in the past 12 months is based on a positive response to the survey question, “In the past 12 months, have you had an appointment with a doctor, nurse, or other health professional by video or by phone?”

c Restricted to adults who reported seeing a doctor or health professional about their health in the past 12 months.

d Diagnosed prediabetes was based on a positive response to the survey question, “Has a doctor or other health professional ever told you that you had prediabetes or borderline diabetes?” and a negative response to the survey question, “Has a doctor or other health professional ever told you that you had diabetes?” Diagnosed diabetes was based on a positive response to the survey question, “Has a doctor or other health professional ever told you that you had diabetes?” irrespective of a prediabetes diagnosis. Adults who responded no to both survey questions were considered to have no prediabetes or diabetes diagnosis. Adults missing complete prediabetes and diabetes diagnosis information were excluded.

e *P* < .05.

f “Other” category is composed of people who identified as non-Hispanic Asian, non-Hispanic American Indian or Alaska Native, other single race, or multiple races.

g Family income was imputed when missing. Family income was reported as a percentage of the FPL based on annual weighted average thresholds published by the US Census Bureau.

h “Private” is adults who reported having any private insurance plan. “Public only” is adults who did not have any private coverage but who reported being covered under Medicaid, Medicare, a state-sponsored health plan, other government program, or military coverage. “Uninsured” is adults who did not report being covered under private health insurance, Medicare, Medicaid, a state-sponsored health plan, other government program, or military coverage.

**Table 2b:** Prevalence of Past 12-Month Telemedicine Use Among Adults With and Without Diagnosed Prediabetes or Diabetes Who Saw a Doctor or Health Professional Within the Past 12 Months: National Health Interview Survey, United States, 2022^a^^,^ b ^,^ c

Characteristic	No prediabetes or diabetes diagnosis (n = 18,037)^d^		Diagnosed prediabetes (n = 2,471)		Diagnosed diabetes (n = 2,814)	
	Unadjusted % (95% CI)	aPR (95% CI)	Unadjusted % (95% CI)	aPR (95% CI)	Unadjusted % (95% CI)	aPR (95% CI)
**Overall**	32.4 (31.5–33.4)	NA	39.6 (37.2–42.1)	NA	39.9 (37.7–42.1)	NA
**Sex**						
Male	29.1 (27.9–30.4)	1 [Ref]	36.8 (33.4–40.3)	1 [Ref]	37.8 (34.8–41.0)	1 [Ref]
Female	35.2 (33.9–36.5)	1.2 (1.2–1.3)^e^	42.0 (38.7–45.3)	1.1 (1.0–1.3)^e^	42.0 (39.0–45.0)	1.1 (1.0–1.2)^e^
**Age, y**						
18**–**44	33.6 (32.3–35.0)	1 [Ref]	45.4 (39.4–51.5)	1 [Ref]	46.0 (38.8–53.3)	1 [Ref]
45**–**64	32.9 (31.4–34.5)	1.0 (0.9–1.0)	39.5 (36.1–43.1)	0.9 (0.8–1.0)	43.4 (40.0–47.0)	1.0 (0.8–1.1)
≥65	29.1 (27.6–30.7)	0.8 (0.8–0.9)^e^	36.2 (33.0–39.6)	0.8 (0.7–1.0)^e^	35.2 (32.5–38.0)	0.8 (0.6–0.9)^e^
**Race and ethnicity**						
White, non-Hispanic	33.7 (32.6–34.9)	1 [Ref]	39.0 (36.1–42.0)	1 [Ref]	39.7 (37.1–42.4)	1 [Ref]
Black, non-Hispanic	28.2 (25.7–30.8)	0.8 (0.7–0.9)^e^	36.3 (31.0–42.0)	0.9 (0.8–1.1)	40.3 (35.0–45.8)	1.0 (0.8–1.1)
Hispanic	30.7 (28.6–32.9)	0.9 (0.8–1.0)^e^	41.6 (35.4–47.9)	1.0 (0.9–1.2)	37.7 (32.4–43.3)	0.9 (0.8–1.1)
Other^f^	31.3 (28.4–34.3)	0.9 (0.8–1.0)^e^	44.6 (37.2–52.2)	1.1 (0.9–1.3)	44.6 (36.7–52.7)	1.1 (0.9–1.3)
**Education**						
No high school diploma or GED	25.1 (22.2–28.2)	1 [Ref]	29.1 (22.6–36.6)	1 [Ref]	28.0 (23.1–33.4)	1 [Ref]
High school diploma or GED	25.5 (23.8–27.2)	1.0 (0.9–1.1)	32.8 (28.5–37.5)	1.2 (0.9–1.5)	36.2 (32.5–40.1)	1.3 (1.0–1.6)^e^
Some college	33.3 (31.7–35.0)	1.3 (1.1–1.5)^e^	42.5 (38.5–46.6)	1.5 (1.2–2.0)^e^	45.0 (40.9–49.1)	1.6 (1.3–2.0)^e^
Bachelor's degree or higher	38.5 (37.1–39.9)	1.5 (1.3–1.7)^e^	47.0 (43.0–51.0)	1.7 (1.3–2.2)^e^	49.2 (44.9–53.6)	1.8 (1.4–2.2)^e^
**Family income, % FPL^g^**						
<100	29.9 (27.2–32.7)	1 [Ref]	44.1 (37.0–51.4)	1 [Ref]	41.1 (35.2–47.3)	1 [Ref]
100 to <200	28.4 (26.5–30.4)	1.0 (0.9–1.1)	37.7 (32.4–43.3)	0.9 (0.7–1.1)	35.4 (31.0–40.1)	0.9 (0.7–1.1)
200 to <400	30.0 (28.4–31.7)	1.0 (0.9–1.1)	37.6 (33.5–41.8)	0.9 (0.7–1.1)	38.4 (34.7–42.3)	1.0 (0.8–1.2)
≥400	35.7 (34.4–37.0)	1.2 (1.1–1.3)^e^	40.9 (37.4–44.5)	1.0 (0.8–1.2)	44.0 (40.1–47.9)	1.1 (0.9–1.3)
**Health insurance^h^**						
Private	33.5 (32.4–34.7)	1 [Ref]	37.9 (35.0–40.9)	1 [Ref]	41.3 (38.3–44.3)	1 [Ref]
Public only	33.9 (32.2–35.6)	1.1 (1.0–1.1)^e^	43.3 (39.3–47.4)	1.2 (1.0–1.3)^e^	39.9 (36.7–43.3)	1.0 (0.9–1.1)
Uninsured	16.1 (13.5–19.0)	0.5 (0.4–0.6)^e^	31.5 (21.2–44.1)	0.7 (0.5–1.1)^e^	18.3 (10.6–29.6)	0.4 (0.2–0.7)
**Urban–rural residence**						
Large central metro	37.5 (35.9–39.2)	1 [Ref]	44.8 (40.3–49.3)	1 [Ref]	44.2 (39.7–48.7)	1 [Ref]
Large fringe metro	35.1 (33.2–37.0)	0.9 (0.8–1.0)^e^	40.9 (36.4–45.5)	0.9 (0.8–1.0)	46.5 (41.8–51.2)	1.0 (0.9–1.2)
Medium and small metro	29.4 (27.7–31.1)	0.8 (0.7–0.8)^e^	39.6 (35.2–44.1)	0.9 (0.7–1.0)	37.3 (34.0–40.7)	0.8 (0.7–0.9)^e^
Nonmetropolitan	22.8 (20.3–25.5)	0.6 (0.5–0.6)^e^	25.8 (20.8–31.5)	0.6 (0.4–0.7)^e^	28.6 (23.8–33.9)	0.6 (0.5–0.8)^e^
**US Census region**						
West	40.1 (37.8–42.4)	1 [Ref]	50.0 (44.7–55.4)	1 [Ref]	45.5 (40.0–51.1)	1 [Ref]
Northeast	36.5 (34.5–38.6)	0.9 (0.8–1.0)^e^	38.5 (33.6–43.7)	0.8 (0.6–0.9)^e^	44.5 (38.9–50.3)	1.0 (0.8–1.2)
Midwest	27.9 (26.0–30.0)	0.7 (0.6–0.7)^e^	34.8 (29.8–40.2)	0.7 (0.6–0.8)^e^	39.5 (35.5–43.6)	0.8 (0.7–1.0)^e^
South	28.3 (26.7–29.9)	0.7 (0.6–0.8)^e^	35.1 (31.4–39.0)	0.7 (0.6–0.8)^e^	35.5 (32.2–39.0)	0.8 (0.7–0.9)^e^

Abbreviations: aPR, adjusted prevalence ratio; FPL, federal poverty level; GED, general educational development certificate; NA, not available.

a Sample sizes (n) are unweighted. Prevalence (%) and associated 95% CIs are weighted and crude. Adjusted prevalence ratios (aPR) were estimated using predictive marginal proportions from logistic regression models controlling for age, sex, and race and ethnicity.

b Telemedicine use in the past 12 months was based on a positive response to the survey question, “In the past 12 months, have you had an appointment with a doctor, nurse, or other health professional by video or by phone?”

c Restricted to adults who reported seeing a doctor or health professional about their health in the past 12 months.

d Diagnosed prediabetes was based on a positive response to the survey question, “Has a doctor or other health professional ever told you that you had prediabetes or borderline diabetes?” and a negative response to the survey question, “Has a doctor or other health professional ever told you that you had diabetes?” Diagnosed diabetes was based on a positive response to the survey question, “Has a doctor or other health professional ever told you that you had diabetes?” irrespective of a prediabetes diagnosis. Adults who responded no to both survey questions were considered to have no prediabetes or diabetes diagnosis. Adults missing complete prediabetes and diabetes diagnosis information were excluded.

e *P* < .05.

f “Other” category is composed of people who identified as non-Hispanic Asian, non-Hispanic American Indian Alaska Native, other single race, or multiple races.

g Family income was imputed when missing. Family income was reported as a percentage of the FPL based on annual weighted average thresholds published by the US Census Bureau.

h “Private” is adults who reported having any private insurance plan. “Public only” is adults who did not have any private coverage but who reported being covered under Medicaid, Medicare, a state-sponsored health plan, other government program, or military coverage. “Uninsured” is adults who did not report being covered under private health insurance, Medicare, Medicaid, a state-sponsored health plan, other government program, or military coverage.

## Discussion

Telemedicine was used by approximately half of US adults diagnosed with prediabetes or diabetes in 2021, with a noticeable decrease in use in 2022. We observed the lowest telemedicine usage among adults without these conditions. Among adults diagnosed with diabetes, we identified persistent disparities by region, urbanicity, insurance status, and educational attainment. Disparities occurred according to these factors among adults diagnosed with prediabetes as well, although female adults with prediabetes were more frequent telemedicine users than male adults.

In 2020, telemedicine use increased substantially due to the COVID-19 pandemic (7). Although nationally representative estimates of telemedicine use among US adults with prediabetes or diabetes before the COVID-19 pandemic are lacking, one previous study reported that 15.0% of US adults with diabetes used broad e-health services (eg, using email to communicate with health providers) in 2013 (8). Our study indicates that telemedicine has become common among US adults with prediabetes or diabetes, with approximately one-third to one-half of adults with these conditions using telemedicine in recent years. However, future studies may be important to characterize patterns and trends in telemedicine use among these populations.

Our study also expands on recent research of telemedicine disparities (9). For example, we observed significantly lower telemedicine use among adults with prediabetes or diabetes living in nonmetropolitan areas, which is concerning since fewer endocrinologists practice in nonmetropolitan areas (10); telemedicine could be leveraged to reduce such health care disparities. Additionally, our results indicated that telemedicine use is less common among adults with lower educational attainment, which may be related to limited digital literacy, access to technologies, or other telemedicine use barriers (11). In efforts to reduce disparities in telemedicine use (12), our study identified groups among adults with prediabetes or diabetes that could benefit from targeted interventions.

Our study has limitations. First, we used self-reported measures that may have been affected by recall and misclassification bias. Second, our data lack specific information on the purpose of the virtual health care visits. Lastly, we were unable to ascertain information on availability and preference for virtual versus in-person health care visits, which limits our ability to contextualize observed disparities.

In conclusion, our findings provide a recent snapshot of the prevalence of telemedicine use among US adults with and without prediabetes or diabetes. Additionally, we identified disparities in telemedicine use among these groups. Further research may elucidate the individual- and system-level barriers associated with telemedicine use among adults with prediabetes or diabetes.
